# Evolution of host specificity in monogeneans parasitizing African cichlid fish

**DOI:** 10.1186/1756-3305-7-69

**Published:** 2014-02-14

**Authors:** Monika Mendlová, Andrea Šimková

**Affiliations:** 1 Department of Botany and Zoology, Faculty of Science, Masaryk University, Kotlářská 2, 611 37, Brno, Czech Republic

**Keywords:** Host specificity, Evolution, Attachment apparatus, Phylogeny, Host predictability, Cichlidae, Monogenea

## Abstract

**Background:**

The patterns and processes linked to the host specificity of parasites represent one of the central themes in the study of host-parasite interactions. We investigated the evolution and determinants of host specificity in gill monogeneans of *Cichlidogyrus* and *Scutogyrus* species parasitizing African freshwater fish of Cichlidae.

**Methods:**

We analyzed (1) the link between host specificity and parasite phylogeny, (2) potential morphometric correlates of host specificity (i.e. parasite body size and the morphometrics of the attachment apparatus), and (3) potential determinants of host specificity following the hypothesis of ecological specialization and the hypothesis of specialization on predictable resources (i.e. host body size and longevity were considered as measures of host predictability), and (4) the role of brooding behavior of cichlids in *Cichlidogyrus* and *Scutogyrus* diversification.

**Results:**

No significant relationships were found between host specificity and phylogeny of *Cichlidogyrus* and *Scutogyrus* species. The mapping of host specificity onto the parasite phylogenetic tree revealed that an intermediate specialist parasitizing congeneric cichlid hosts represents the ancestral state for the *Cichlidogyrus*/*Scutogyrus* group. Only a weak relationship was found between the morphometry of the parasites’ attachment apparatus and host specificity. Our study did not support the specialization on predictable resources or ecological specialization hypotheses. Nevertheless, host specificity was significantly related to fish phylogeny and form of parental care.

**Conclusions:**

Our results confirm that host specificity is not a derived condition for *Cichlidogyrus*/*Scutogyrus* parasites and may reflect other than historical constraints. Attachment apparatus morphometry reflects only partially (if at all) parasite adaptation to the host species, probably because of the morphological similarity of rapidly evolved cichlids (analyzed in our study). However, we showed that parental care behavior of cichlids may play an important role linked to host specificity of *Cichlidogyrus*/*Scutogyrus* parasites.

## Background

Host specificity is considered to be a result of various factors including phylogenetic, physiological and ecological aspects [1-5]. Most parasites exhibit at least some degree of host specificity or host preference [6,7]; however, the basics of features governing the evolution of host specificity are not yet fully understood. The class Monogenea mainly includes ectoparasites with a direct life cycle, high morphological diversity, and high species richness. These parasites are highly host specific when compared to other groups of parasites [8-10]. For these reasons, the genera of Monogenea are often selected as suitable models for studying the patterns and processes connected with the evolution of parasite specialization, resulting in host specificity [1,11,12]. It has also been suggested that host specificity can be considered as a prerequisite of parasite speciation [13,14]. Finally, host specificity is supposed to be associated with adaptive specialization [15]. However, high host specificity does not necessarily reflect a historical association between hosts and parasites, because host-parasite systems that evolve as a result of host switching may also show a high degree of host specificity [13]. In host specific monogeneans, evolution by intrahost duplication and/or host switching was shown [12,16,17]. Thus, host specificity may more likely describe current host-parasite relationships, which may or may not reflect macroevolutionary history [14].

Host specificity is traditionally expressed as the number of host species exploited by a given parasite species and depends on the number of host species in/on which the parasite may exist [18]. Taking into account the number of host species and host phylogenetic relatedness, parasites may occur on a single host species, on congeneric host species, on phylogenetically closely related non-congeneric hosts, or on phylogenetically unrelated host species [1,12]. Host specificity may be determined by host predictability following the hypothesis of specialization on predictable resources [19]. This hypothesis postulates that organisms tend to specialize on stable resources, which minimizes their extinction risks [19]. The most predictable resources for parasites are the largest, long-lived, and more abundant hosts or hosts on top of the food chain [3,12,20,21]. This hypothesis was previously tested and confirmed in congeneric monogeneans parasitizing the gills of marine Sparidae [1] and freshwater Cyprinidae fish [12,21]. In addition, following the hypothesis of ecological specialization, host specificity may be linked to parasite distribution [11,22]. The basic assumption of this hypothesis is that species exploiting more resources are more widespread and more abundant in nature than species restricted to a narrow range of resources [23,24]. When applying the hypothesis of ecological specialization to parasites, generalists using a wide range of host species should be more abundant on their hosts than specialists. This hypothesis was confirmed in gill monogeneans of the species *Dactylogyrus*[11,12], but it was not supported for adult nematodes in terrestrial mammal hosts [22].

The morphology of the attachment apparatus of parasites (especially in monogeneans) may have an important role in specialization and adaptation to their hosts [12,21,25,26]. Jarkovský *et al*. [27] pointed out that more similarities in the attachment apparatus (i.e. haptor) of congeneric monogeneans are found within specialist infracommunities than within generalist infracommunities, but that the similarity in copulatory organs of specialist parasites seems to follow a random pattern. Specialists have an attachment apparatus closely adapted to their host and the similarity in attachment apparatus is the result of specialization to the host species or microhabitats within the host [25].

From an evolutionary point of view, cichlid fish represent a very interesting model due to their extensive and rapid radiation and diversification. Cichlids have a highly organized system of reproductive activities and brooding behavior concerning their parental care. A few studies using molecular phylogenetic analyses to investigate the coevolution of cichlids and their host specific gill monogeneans have been performed [17,28,29]. However, the potential correlates and determinants of host specificity in cichlid monogeneans have not yet been investigated. African cichlids are parasitized by five genera of monogeneans belonging to Dactylogyridea, i.e. *Cichlidogyrus* Paperna, 1960; *Scutogyrus* Pariselle & Euzet, 1995; *Onchobdella* Paperna, 1968; *Enterogyrus* Paperna, 1963; and *Urogyrus* Bilong Bilong, Birgi & Euzet, 1994. Among them, the *Cichlidogyrus* genus represents the most diversified species group, i.e. 87 species of *Cichlidogyrus* are known from cichlids living in Africa, the Levant, and Madagascar [30-35]. A further 7 gill parasite species belong to the *Scutogyrus* genus and 8 gill parasite species belong to *Onchobdella*[30,34]. Phylogenetic analyses based on molecular data showed that *Cichlidogyrus* and *Scutogyrus* form a monophyletic group (i.e. *Scutogyrus* has the nested position in *Cichlidogyrus* phylogeny), whilst *Onchobdella* is closely related to endoparasitic *Enterogyrus*[29]. From 54 *Cichlidogyrus* and *Scutogyrus* species infesting the tilapias (i.e. *Tilapia*, *Sarotherodon* and *Oreochromis* species), only 18 species are generalists infecting two or more host species, but no *Cichlidogyrus* species infects cichlids with different parental care behavior, i.e. mouthbrooders and substrate-brooders [28].

The aim of this study was to describe the patterns connected with host specificity in monogenean parasites of the *Cichlidogyrus*-*Scutogyrus* group parasitizing West African Cichlidae. We focused on (1) the morphometric correlates of host specificity, hypothesizing the role of haptor morphology in specialization and adaptation, (2) parasite abundance following the hypothesis of ecological specialization, and (3) the determinants of host specificity following the hypothesis of specialization on predictable resources (i.e. hosts) and on the basis of the assumption that parental care behavior in cichlids represents an important factor linked to host specificity. In addition, we investigated whether host specificity is constrained by parasite phylogeny and, if so, whether host specificity represents an ancestral or derived state for parasites of the *Cichlidogyrus-Scutogyrus* group.

## Methods

### Parasite data

**Figure 1:**
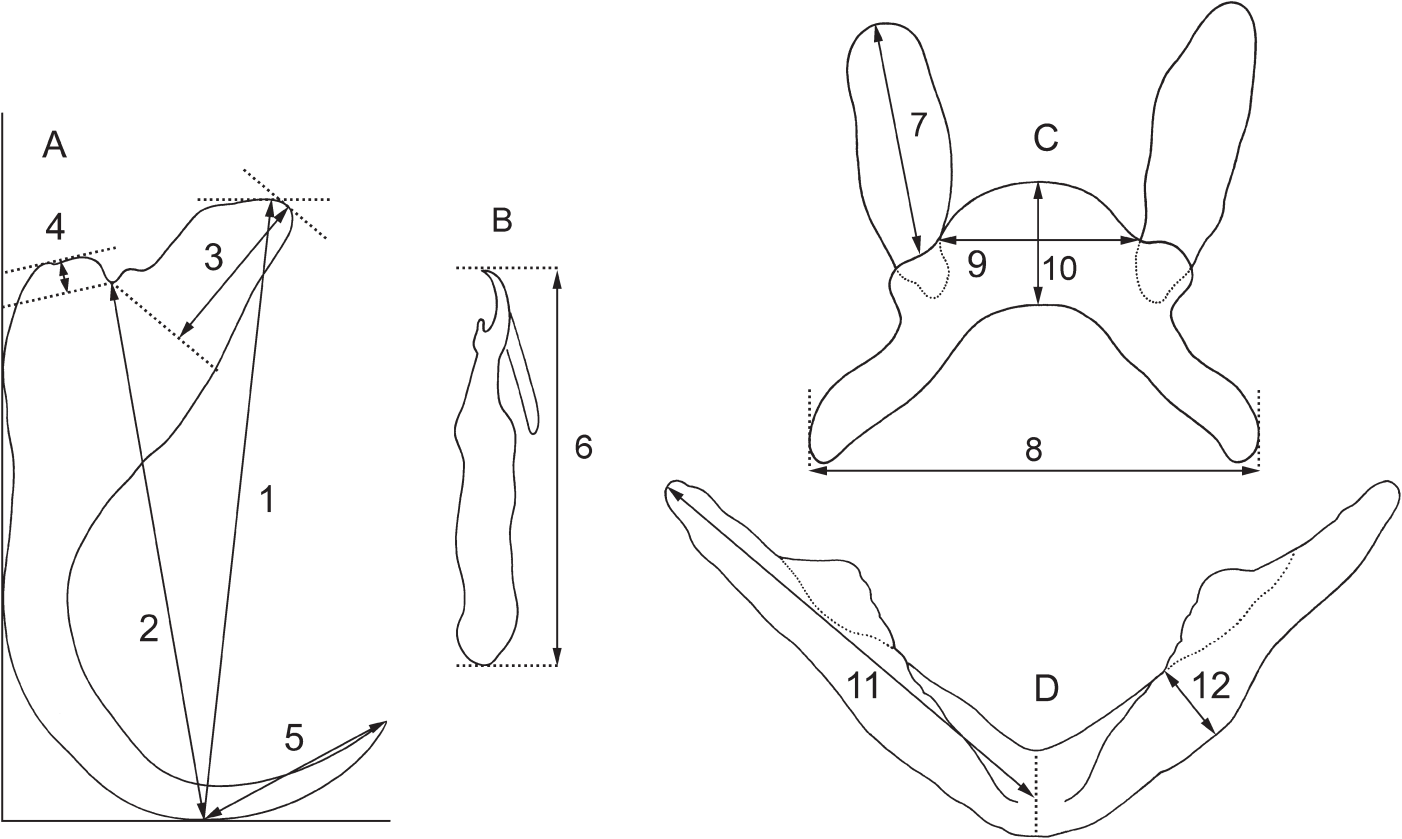
**Measurements of the sclerotized structures of the haptor used in this study. (A)** Anchor: 1 – total length, 2 – length to notch, 3 – length of inner root, 4 – length of outer root, 5 – length of point; **(B)** Marginal hooks: 6 – total length; **(C)** Dorsal bar: 7 – length of auricles, 8 – total width, 9 – distance between auricles, 10 – thickness; **(D)** Ventral bar: 11 – branch length, 12 – maximum width.

Six cichlid species i.e. *Hemichromis fasciatus* Peters, 1857; *Hemichromis letourneuxi* Sauvage, 1880; *Tilapia guineensis* (Bleeker, 1862); *Oreochromis niloticus* (Linnaeus, 1758); *Sarotherodon galilaeus* (Linnaeus, 1758); and *Tylochromis intermedius* (Boulenger, 1916) were collected and investigated for the presence of gill parasites, *Cichlidogyrus* and *Scutogyrus* species (Monogenea) during an extensive field work study carried out at the Niokolo Koba National Park (Senegal, Africa) in 2004–2008. Fish were examined by standard parasitological methods described in [36]. Monogeneans were removed from the gills of fish, placed on slides, fixed in a mixture of glycerine and ammonium picrate [37], covered by a coverslip, and identified using a light microscope equipped with phase contrast and digital image analysis (Micro Image 4.0 for Windows, Olympus Optical Co., Hamburg, Germany). The sclerotized parts of the parasite attachment organ i.e. haptor (see Figure 1) and reproductive organs (vagina and copulatory organ) were used for parasite determination. A total of 19 *Cichlidogyrus* and 2 *Scutogyrus* species were collected from the gills of 86 cichlid specimens. This dataset of parasite species was supplemented including another 6 *Cichlidogyrus* species and one *Scutogyrus* species for which the molecular data were available [28]. Altogether, data on 28 monogenean parasite species including 25 *Cichlidogyrus* species and 3 *Scutogyrus* species were included in this study. All of them have valid species status.

### Phylogenetic distances

Molecular analyses confirmed the species status of all *Cichlidogyrus* and *Scutogyrus* included in our study (Mendlová *et al*. [17]). Phylogenetic information for parasites and hosts were obtained from the study of Mendlová *et al*. [17] and included in this study. The partial SSU rDNA and entire ITS1 regions for *Cichlidogyrus* and *Scutogyrus* species and the partial region of the cytochrome *b* gene for cichlid fishes were used for phylogenetic analyses.

**Figure 2:**
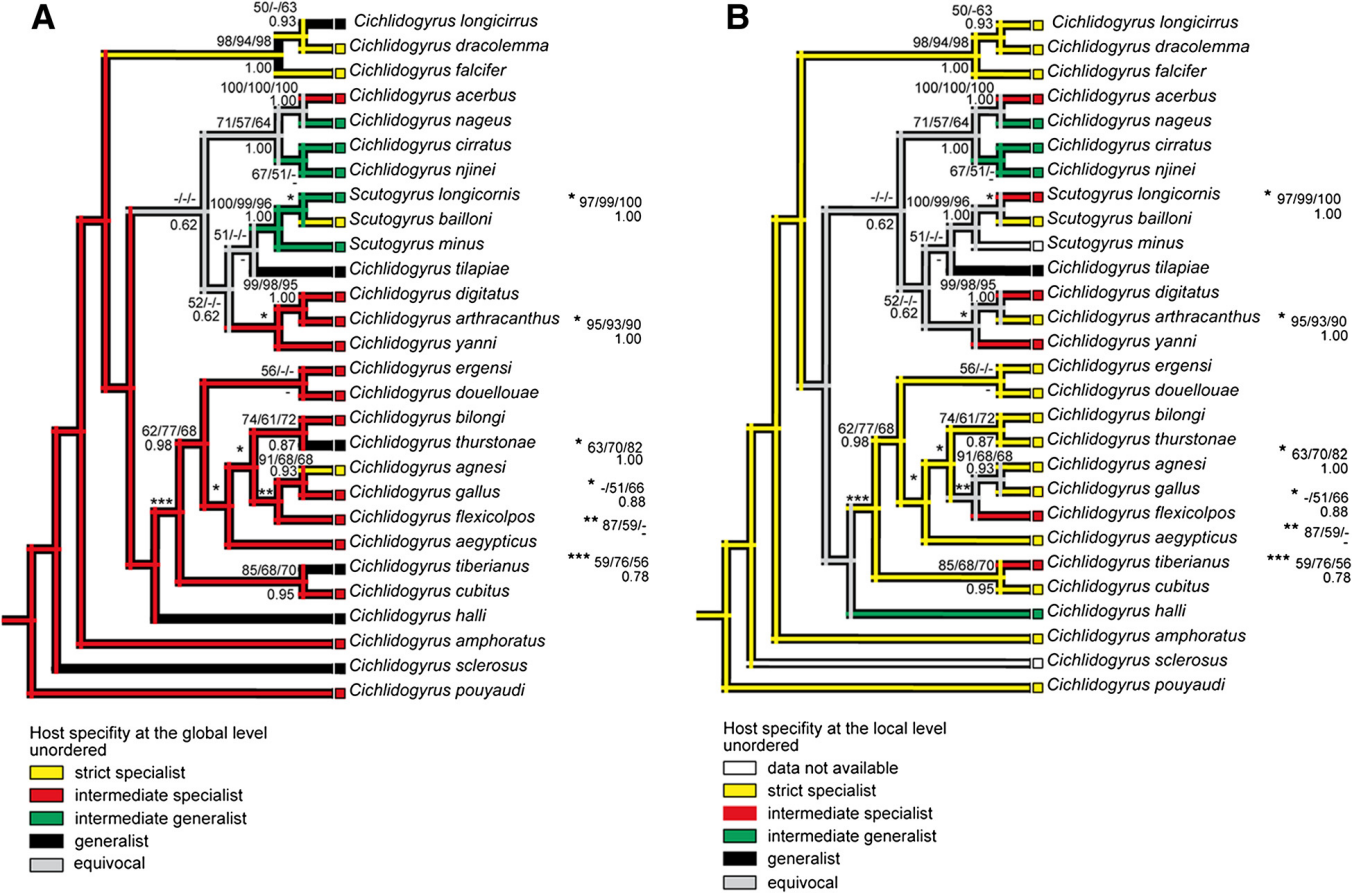
**Mapping of host specificity onto the parasite phylogenetic tree. (A)** Mapping of the index of host specificity at the global level onto the parasite phylogenetic tree; **(B)** mapping of the index of host specificity at the local level onto the parasite phylogenetic tree. Numbers along branches indicate bootstrap proportions resulting for ME/MP/ML analyses (above branches) and posterior probabilities resulting from BI analysis (below branches).

ModelTest [38] was applied to select the most appropriate substitution model of nucleotide evolution for each data set using hierarchical likelihood ratio tests (hLRTs). ML distances based on the selected model (TrN + G) were calculated as the measure of phylogenetic distances between species of the *Cichlidogyrus* and *Scutogyrus* group. In the case of Cichlidae, ML distances corresponding to the GTR + I + G model were calculated as the measure of phylogenetic distances between fish species. Bootstrap values for minimum evolution (ME), maximum parsimony (MP), maximum likelihood (ML), and posterior probabilities for BI (Bayesian inference) obtained from phylogenetic analyses by Mendlová *et al*. [17] are presented in Figure 2. Principal coordinate analysis (PCoA) with corrections for negative eigenvalues was performed on the phylogenetic distance matrices for fish or parasites. This eigenvector method was shown to be efficient for representing phylogenetic inertia [39,40]. The principal coordinates (PCs) were computed in DistPCoA written in FORTRAN [41]. The computed PCs represented the phylogenetic variance. The non-significant PCs were eliminated using the Broken Stick model [42]. Significant PCs were retained for regressions (see below).

### Delimitation of host specificity

First, host specificity was expressed at the local level using our data from the field study in Senegal and the host-parasite records for Senegal using Pariselle and Euzet [30]. We applied the simple classification of parasites as specialists infecting a single host species or generalists infecting more than one host species [43]. Next, host specificity was defined at the global level on the basis of all host-parasite records for African cichlids [30-35]. The list of monogenean species used in this study and their host species found at the local and global levels are given in Additional file 1. Host specificity at the global level was used for the analyses of the morphometric correlates and determinants of host specificity (see below). Because a very low number of parasite species were found to be strict specialists (i.e. parasite species infecting a single host species) at the global level, we were unable to perform statistical analyses (i.e. the low number of parasites in the specialist group would have produced weak statistical correlations). Therefore, we adapted the delimitation of host specificity defined for *Dactylogyrus* species by Šimková *et al*. [12] in order to separate the groups of specialists and generalists in our study, i.e. we considered parasite species infecting one host species or infecting congeneric host species as specialists (this last group of parasites is also termed as intermediate specialists); all other parasite species were considered as generalists. In addition, we defined the index of host specificity (IS) by adapting the approach of Desdevises *et al*. [1] and Šimková *et al*. [12] to our host-parasite system. Thus, on the basis of the extent of host specificity and the phylogenetic relationships among their host species, cichlid parasite species were divided into four groups as follows: (1) strict specialists infecting only one host species; (2) intermediate specialists living on two or more congeneric host species; (3) intermediate generalists infecting noncongeneric cichlid species of Tilapiini; and (4) generalists infecting noncongeneric cichlid species of at least two different tribes (i.e. Tilapiini, Haplochromini, Chromidotilapiini and Hemichromini in our study). Higher IS represents a decrease in host specificity towards generalist forms. The index of host specificity was calculated at the local and global levels. Finally, host range as the total number of cichlid host species living in Africa infected by a given parasite species was recorded. Data on host specificity for all analyzed parasite species are shown in Additional file 2.

### Link between host specificity and parasite phylogeny

We investigated whether host specificity is linked to parasite phylogeny. Multiple stepwise regression analysis with backward elimination was used to assess the relationship between parasite phylogeny (expressed as phylogenetic PCs that are linearly independent of one another by definition) and host specificity expressed as IS at the global level or host range.

Analyses of the evolution of host specificity were performed to assess whether host specificity is a derived or an ancestral condition. Indeces of host specificity defined at the global and local levels for 28 *Cichlidogyrus* and *Scutogyrus* species were mapped onto the fully-resolved minimum evolution parasite tree inferred from the phylogenetic analysis of combined SSU rDNA and ITS1 sequence data. The mapping was performed using MacClade version 4.0.1 with Farris optimization [44]. In addition, to test whether host specificity is a derived condition, IS was regressed against the number of nodes separating each parasite species from the root of the phylogenetic tree.

### Link between host specificity and morphological adaptation

Morphometric measurements of the haptor of 28 *Cichlidogyrus* and *Scutogyrus* species parasitizing cichlid fishes in Africa were used in this study. Parasite body size and the following 23 morphometric variables of the haptor were obtained from published species descriptions [31-34,45-59] and our unpublished data: *total length of ventral* and *dorsal anchors*, *length to notch of ventral* and *dorsal anchors*, *length of point of ventral* and *dorsal anchors*, *length of inner root of ventral* and *dorsal anchors*, *length of outer root of ventral* and *dorsal anchors*, *total lengths of marginal hooks of the first to the seventh pair* (*MH1-7*), *branch length* and *maximum width of ventral transverse bar*, *length* and *distance between auricles of dorsal transverse bar*, and *total width* and *thickness of dorsal transverse bar* (Figure 1). Marginal hooks were numbered following Pariselle and Euzet [54]: MH1: medio-ventral, MH2: ventral associated with ventral anchors, MH3 + MH4: latero-dorsal, MH5-MH7: latero-ventral. The maximum values of all morphometric characters of haptor and maximum body size obtained from parasite species descriptions were considered in the analyses, as applied in Šimková *et al*. [12]. Morphometric variables of the haptor were log-transformed prior to regression analyses.

Multiple stepwise regression analyses with backward elimination were performed to determine whether morphometric characters of the haptor are linked to host specificity expressed as global IS or host range. Bonferroni correction was applied for multiple tests.

### Determinants of host specificity

The potential determinants of host specificity including fish phylogeny, body size, longevity, and parental care behavior were tested using multiple stepwise regression analyses with backward elimination. First, analyses were performed separately for specialists and generalists defined at the global level (see above). Next, host specificity expressed as global IS or host range (dependent variable) were regressed against fish variables. Concerning parasites infecting more than one host species, the mean host size and mean longevity of all available host species were used in the analyses. Using body size and the longevity of preferred host species as applied in the studies of Desdevises *et al*. [1] and Šimková *et al*. [12] was not possible in our study because of a lack of knowledge on parasite abundance in many host species (this did not allow us to determine the preferred host for generalist parasite species) and a lack of knowledge on cichlid longevity in some potentially preferred host species. Data with respect to fish body size expressed as maximum standard length (in cm), longevity, and parental care behavior were obtained from Paugy *et al*. [60], Stiassny *et al*. [61], and Froese and Pauly [62]. For some fish species, data on longevity were not available. In such cases we used the longevity of the most closely phylogenetically related congeneric species. Cichlid species were separated into three groups on the basis of their parental care behavior: (1) mouthbrooders, (2) substrate-brooders, and (3) both types of behavior.

Following the hypothesis of ecological specialization applied to the host-parasite system, we tested whether host specificity expressed by the index of host specificity or host range is linked to parasite distribution measured by parasite abundance. The parasite abundance of a given parasite species was calculated as the total number of parasite specimens on all investigated fish specimens infected by this parasite species divided by the number of investigated fish specimens.

## Results

### Host specificity in species of the *Cichlidogyrus*/*Scutogyrus* group

At the global level of investigation, a total of 17 *Cichlidogyrus* and *Scutogyrus* species were identified as specialists (including 4 strict specialists and 13 intermediate specialists following the classification of Šimková *et al*. [12]); 11 parasite species were identified as generalists. At the local level, 21 species were specialists (15 strict specialists and 6 intermediate specialists) and only 5 species were recognized as generalists. The host range of *Cichlidogyrus* and *Scutogyrus* species parasitizing African cichlids varied from 1 (for *C. agnesi*, *C. dracolemma*, *C. falcifer* and *S. bailloni*) to 21 (for *C. tilapiae*).

### Link between host specificity and parasite phylogeny

Five PCs made a significant contribution to the total phylogenetic variance: PC1 represented 12.1%; PC2, 9.6%; PC3, 8.2%; PC4, 6.5%; and PC5, 4.8% of the total phylogenetic variance. No significant relationships were found between PCs extracted from the parasite phylogenetic distance matrix and host specificity expressed as global IS, local IS, or host range (*P* > 0.05), which indicates no statistical link between parasite phylogeny and host specificity.

Nevertheless, the mapping of the index of host specificity at the global level onto the parasite phylogenetic tree (Figure 2A) revealed that being an intermediate specialist (i.e. infecting congeneric host species) represents the ancestral state for *Cichlidogyrus/Scutogyrus* species. In addition, this character state was recognized for approximately half of the parasite species included in the phylogenetic tree. Several independent changes to the strict specialist state or generalist states were recognized from this mapping. Nevertheless, all intermediate generalists were situated within the same large clade, the ancestral origin of which was unresolved. When the mapping of the index of host specificity was performed at the local level (Figure 2B), a strict specialist was recognized as the ancestral state for *Cichlidogyrus*/*Scutogyrus* parasites. Intermediate specialists, intermediate generalists, and generalists represented the derived states of host specificity for these parasites.

The mapping of host specificity at the global and local levels showed that specialists (i.e. strict specialists and intermediate specialists) are equally presented among the derived and basal species. No statistical link was found between global or local IS and the number of nodes deduced from the parasite phylogenetic tree (*P* > 0.05). These results confirm that being specific is not a derived condition in *Cichlidogyrus*/*Scutogyrus* parasites.

### Link between host specificity and morphological adaptation

Positive relationships were found between parasite body size and morphological characters of the attachment apparatus (Table 1) for all parasite species (i.e. in the analyses without separating specialists and generalists) and also for both specialists and generalists in separated analyses. Parasite body size was not related to PCs extracted from parasite phylogeny (Table 2). Multiple regressions of parasite body size and morphometric measurements of the haptor against the PCs revealed significant relationships between parasite phylogeny and haptor morphometry. When analyzing all species, *total length*, *length to notch* and *outer root of both dorsal and ventral* anchors, and *maximum width of ventral bar* were significantly related to parasite phylogeny after correcting morphometric measurements of the haptor for parasite body size (Table 2). When separate analyses were performed for specialists and generalists defined at the global level, the morphometry of dorsal and ventral anchors (*total length* and *length to notch*), and the *thickness of dorsal bar* of specialists were significantly related to PCs. Concerning generalist parasites, the morphometry of the dorsal anchors (*total length* and *length of outer root*) and ventral anchors (*length of point*) were significantly related to PCs (Table 2). Simple linear regression revealed a significant relationship between host specificity expressed by host range and *total length of dorsal anchors* (n = 28, R^2^ = 0.172, b = 32.470, *P* = 0.016) and *inner root of dorsal anchors* (n = 28, R^2^ = 0.181, b = 22.490, *P* = 0.014) both of them being statistically significant also after Bonferroni correction. When host specificity was expressed by global IS, significant relationships between host specificity and *length to notch of dorsal* (n = 28, R^2^ = 0.202, b = 8.736, *P* = 0.009) and *ventral anchors* (n = 28, R^2^ = 0.144, b = 8.092, *P* = 0.026), *length of marginal hooks of the 1st pair* (n = 28, R^2^ = 0.192, b = −3.992, *P* = 0.011), and *thickness of dorsal bar* (n = 24, R^2^ = 0.141, b = 3.399, *P* = 0.040) were found. However, only the relationships between global IS and a) *length to notch of dorsal anchors* and b) *length of marginal hooks of the 1st pair* were statistically significant after Bonferroni correction.

**Table 1:** Relationships between parasite body size (in log) and measurements of the attachment apparatus (in log)

		**All parasites**				**Generalists**				**Specialists**			
**Dependent variable**		**n**	**b**	***P***	**R**^**2**^	**n**	**b**	***P***	**R**^**2**^	**n**	**b**	***P***	**R**^**2**^
Dorsal anchors	Total length	28		ns		11		ns		17		ns	
	Length to notch	28		ns		11		ns		17		ns	
	Outer root	28	0.717	0.037	0.124	11		ns		17	0.750	0.017	0.326
	Inner root	28		ns		11		ns		17	0.420	0.043	0.245
	Point	28		ns		11		ns		17		ns	
Ventral anchors	Total length	28		ns		11		ns		17		ns	
	Length to notch	28		ns		11		ns		17		ns	
	Outer root	28	0.610	0.043	0.116	11		ns		17	0.777	0.019	0.316
	Inner root	28	0.515	0.002	0.282	11		ns		17	0.598	0.019	0.314
	Point	28	0.296	0.001	0.312	11	0.298	0.049	0.294	17	0.263	0.026	0.289
Marginal hooks	MH1	28		ns		11		ns		17		ns	
	MH2	28	0.172	0.015	0.178	11		ns		17		ns	
	MH3	28	0.609	0.005	0.239	11		ns		17	0.756	0.009	0.377
	MH4	28	0.543	0.007	0.220	11		ns		17	0.723	0.006	0.400
	MH5	28	0.431	0.021	0.158	11		ns		17	0.665	0.008	0.386
	MH6	28	0.469	0.015	0.176	11		ns		17	0.687	0.007	0.391
	MH7	28	0.538	0.008	0.214	11		ns		17	0.685	0.008	0.380
Dorsal bar	Length of auricles	28	0.897	<0.001	0.383	11		ns		17	0.989	<0.001	0.572
	Total width	28	0.443	0.003	0.258	11	0.575	0.047	0.299	17		ns	
	Distance between auricles	26		ns		10		ns		16	0.388	0.043	0.261
	Thickness	24		ns		9		ns		15		ns	
Ventral bar	Branch length	28	0.307	0.006	0.229	11		ns		17	0.363	0.004	0.439
	Maximum width	23	1.329	0.008	0.254	10		ns		13	1.546	0.009	0.480

Parasite body size was used as independent variable. ns - non-significant relationship, b - the slope of regression, R^2^ - the regression coefficient.

**Table 2:** Relationships between PCs and parasite body size (in log) or measurements of the attachment apparatus (in log)

		**All parasites**					**Generalists**					**Specialists**				
**Dependent variable**			**n**	**b**	***P***	**R**^**2**^**( *P *)**		**n**	**b**	***P***	**R**^**2**^**( *P *)**		**n**	**b**	***P***	**R**^**2**^**( *P *)**
Parasite body size			28		ns			11		ns			17		ns	
Dorsal anchors	Total length	PC3	28	−1.408	<0.001	0.336 <0.001	PC1	11	1.290	0.016	0.713 (0.008)	PC3	17	−1.954	<0.001	0.589 (<0.001)
							PC2		2.015	0.003						
							PC4		−1.365	0.011						
	Length to notch	PC3	28	−1.967	<0.001	0.620 (<0.001)		11		ns		PC3	17	−2.141	<0.001	0.687 (<0.001)
	Outer root	PC3	28	2.887	0.003	0.434 (<0.001)	PC5	11	−8.250	0.002	0.630 (0.002)		17		ns	
		PC5		−4.060	0.001											
	Inner root		28		ns			11		ns			17		ns	
	Point		28		ns			11		ns			17		ns	
Ventral anchors	Total length	PC1	28	0.914	<0.001	0.483 (<0.001)		11		ns		PC3	17	−0.971	0.005	0.427 (0.005)
		PC4		−0.787	0.003											
	Length to notch	PC1	28	−0.969	0.002	0.296 (0.002)		11		ns		PC3	17	−1.167	0.005	0.420 (0.005)
	Outer root	PC3	28	3.433	<0.001	0.390 (<0.001)		11		ns			17		ns	
	Inner root		28		ns			11		ns			17		ns	
	Point		28		ns		PC2	11	1.324	0.006	0.631 (0.008)		17		ns	
							PC4		−1.113	0.008						
Marginal hooks	MH1		28		ns			11		ns			17		ns	
	MH2		28		ns			11		ns			17		ns	
	MH3		28		ns			11		ns			17		ns	
	MH4		28		ns			11		ns			17		ns	
	MH5		28		ns			11		ns			17		ns	
	MH6		28		ns			11		ns			17		ns	
	MH7		28		ns			11		ns			17		ns	
Dorsal bar	Length of auricles		28		ns			11		ns			17		ns	
	Total width		28		ns			11		ns			17		ns	
	Distance between auricles		26		ns			10		ns			16		ns	
	Thickness		24		ns			9		ns		PC3	15	−2.246	0.002	0.537 (0.002)
Ventral bar	Branch length		28		ns			11		ns			17		ns	
	Maximum width	PC5	23	-4.718	0.003	0.361 (0.003)		10		ns			13		ns	

Measurement of the attachment apparatus corrected for parasite body size. ns - non-significant relationship, b - the slope of regression, R^2^ - the regression coefficient.

### Determinants of host specificity

First, we investigated whether host specificity was linked to parasite abundance following the hypothesis of ecological specialization. No significant relationship was found between host specificity (expressed by global IS, local IS, or global host range) and parasite abundance (*P* > 0.05) which suggests that generalists infecting a wide range of host species are not more abundant on their hosts than specialists restricted to a narrow range of host species.

Next, we investigated whether host specificity is linked to fish phylogeny. We performed principal coordinate analyses as were previously applied in the case of cichlid parasites. Five FishPCs contributed significantly to the total phylogenetic variance and thus represented fish phylogeny, FishPC1 represented 15.6%; FishPC2, 9.6%; FishPC3, 6.6%; FishPC4, 5.5%; and FishPC5, 5.0% of the total phylogenetic variance. No significant relationship was found between these fish phylogeny and host specificity (expressed as global IS or host range). In the next step, we tested a potential link between fish phylogeny and investigated fish traits (representing potential determinants of host specificity) i.e. host body size, longevity and form of parental care. Significant relationships were found between FishPCs and a) host body size and b) host longevity; however, no significant relationship was found between FishPCs and the form of parental care (*P* > 0.05) (Table 3).

**Table 3:** Link between host phylogeny and potential determinants of host specificity

	**Independent variable (FishPCs)**				
**Dependent variable**		**n**	**b**	***P***	**R**^**2**^**( *P *)**
Host body size	FishPC1	26	−1.850	<0.001	0.717 (<0.001)
	FishPC5		−1.595	<0.001	
Longevity	FishPC1	26	−45.556	<0.001	0.867 (<0.001)
	FishPC2		16.168	<0.001	
	FishPC3		−35.804	<0.001	
	FishPC4		26.132	<0.001	
	FishPC5		−42.710	<0.001	
Parental care		26		ns	

ns - non-significant relationship, b - the slope of regression, R^2^ - the regression coefficient.

The relationships between parasite body size or haptor morphometric variables and potential determinants of host specificity were analyzed separately for specialists and generalists defined at the global level. No significant relationship between parasite body size and host body size corrected for phylogeny in specialists and generalists was found (Table 4). Considering specialists, *total length of anchors*, *length of marginal hooks of the 2nd pair* and *maximum width of ventral bar* were, after correcting for parasite body size and phylogeny, significantly related to fish phylogeny and form of parental care. In addition, *marginal hooks of the 2nd pair* and *maximum width of ventral bar* of specialists were also significantly related to cichlid longevity when corrected for fish phylogeny. However, considering generalists, haptor morphometry (i.e. six morphometric variables representing the dorsal and ventral anchors, the marginal hooks of the 1st pair, and the dorsal and ventral bars) was significantly related to mean fish body size. In addition, significant relationships were found between the *length of inner root of the dorsal anchors* and cichlid longevity when corrected for fish phylogeny, and between a) the *lengths of the marginal hooks (all pairs except of 2nd pair)* and b) *width of ventral bar* and the form of parental care (Table 4).

**Table 4:** Determinants of host specificity

		**Independent variables**									
		**Generalists**					**Specialists**				
**Dependent variable**			**n**	**b**	***P***	**R2**		**n**	**b**	***P***	**R2**
Parasite body size		FishPC4	9	−0.483	0.033	0.429		17		ns	
Dorsal anchors	Total length		9		ns		FishPC4	17	0.236	0.007	0.355
							Parental care	17	0.072	0.017	0.281
	Length to notch		9		ns			17		ns	
	Outer root		9		ns			17		ns	
	Inner root	Longevity	9	0.112	0.010	0.589		17		ns	
	Point	Host body size	9	0.779	0.029	0.448		17		ns	
Ventral anchors	Total length		9		ns			17		ns	
	Length to notch		9		ns			17		ns	
	Outer root		9		ns			17		ns	
	Inner root	FishPC3	9	−1.055	0.031	0.436		17		ns	
	Point	Host body size	9	0.272	0.030	0.443		17		ns	
Marginal hooks	MH1	Parental care	9	−0.068	0.021	0.492		17		ns	
		Host body size	9	0.696	0.043	0.390					
	MH2	FishPC3	9	−0.807	0.004	0.668	FishPC3	17	−0.714	0.001	0.495
		FishPC4	9	−0.310	0.005	0.661	FishPC4	17	−0.217	0.001	0.487
							Parental care	17	−0.049	0.050	0.182
							Longevity	17	−0.358	0.005	0.382
	MH3	Parental care	9	−0.137	0.028	0.452		17		ns	
	MH4	Parental care	9	−0.126	0.027	0.461		17		ns	
	MH5	Parental care	9	−0.113	0.013	0.553		17		ns	
	MH6	Parental care	9	−0.122	0.011	0.573		17		ns	
	MH7	Parental care	9	−0.129	0.032	0.436		17		ns	
Dorsal bar	Length of auricles	Host body size	9	1.707	0.037	0.413		17		ns	
	Total width		9		ns			17		ns	
	Distance between auricles	Host body size	8	0.931	0.009	0.659		16		ns	
	Thickness		7		ns			15		ns	
Ventral bar	Branch length	Host body size	9	1.072	0.009	0.596		17		ns	
	Maximum width	Parental care	8	−0.229	0.049	0.418	FishPC3	13	−4.158	0.017	0.363
							FishPC4	13	−0.703	0.040	0.270
							FishPC5	13	−1.020	0.018	0.358
							Parental care	13	−0.272	0.015	0.381
							Longevity	13	−1.368	0.024	0.326

Measurements of the attachment apparatus corrected for parasite body size and phylogeny. ns - non-significant relationship, b - the slope of regression, R^2^ - the regression coefficient.

Finally, to analyze whether host specificity is determined by host predictability following the hypothesis of specialization on predictable resources and/or linked to parental care behavior, host specificity (expressed by global IS, local IS and host range) was regressed against the following fish traits: body size, longevity, parental care behavior, and phylogeny. A significant positive relationship was found between host range and cichlid longevity when corrected for fish phylogeny. Host specificity expressed as global IS was significantly related to a) fish phylogeny, b) longevity corrected for fish phylogeny, and c) form of parental care. Host specificity expressed as local IS was significantly related to a) fish phylogeny, and b) form of parental care (Table 5).

**Table 5:** Link between host specificity and host traits considered as potential determinants of host specificity

	**Independent variables**				
**Host specificity**	**Statistically significant variables**	**n**	**b**	***P***	**R**^**2**^**( *P *)**
Host range	Longevity	26	9.231	<0.001	0.460 (<0.001)
IS global	Longevity	26	1.078	0.004	0.600 (<0.001)
	Parental care		0.701	0.001	
	FishPC1		−7.744	0.003	
	FishPC5		−8.481	0.001	
IS local	Parental care	26	0.898	< 0.001	0.624 (<0.001)
	FishPC4		−2.951	< 0.001	

Host specificity expressed by global IS, local IS, or host range. b - the slope of regression, R^2^ - the regression coefficient.

## Discussion

The delimitation of host specificity is a crucial concern in ecological and evolutionary studies. As previously suggested (e.g. Poulin *et al*. [63]) and also implied from our study, host specificity should be evaluated with respect to how closely related host species are, or whether representatives of particular parasite species exploit the same or different host species across their geographic range. The degree of host specificity for a given parasite species may differ when considering different scales of investigation [64]. Some parasite species may show high host specificity on a local scale and exhibit lower host specificity (i.e. they are generalists) on a global scale, or vice versa, whilst other parasite species may exhibit the same host specificity at both levels i.e. they are either specialists or generalists [63,64]. In our study, we found that the degree of host specificity of many *Cichlidogyrus* and *Scutogyrus* species differs at the global and local levels of investigation. At the local level, many *Cichlidogyrus* species and one *Scutogyrus* species were strictly host specific. However, at the global level, these parasite species were mainly recorded in congeneric hosts or, in some rare cases, in phylogenetically unrelated species. The difference in host specificity between local and global levels of investigation was also previously observed in *Dactylogyrus* species parasitizing cyprinid fish [12]. Such a difference could primarily result from the geographical distribution of suitable host species (i.e. the absence of some host species or rare occurrence of some host species in a given locality) and may be explained by the isolation of parasite populations or the aggregated distribution of parasites (some host species or some populations of the same host species are more often parasitized than others). Variation in host specificity across the geographic range was also found in other monogenean species parasitic on fish of the Centrarchidae in Nebraska by Collins and Janovy [65]. They showed that the parasites are more host specific at the local level than might be inferred from published host-parasite records and parasite species do not necessarily colonize all supposedly receptive host species even when these host species are present. The problem of delimiting host specificity on different spatial scales warns against using data from local investigations in evolutionary studies.

### Host specificity constrained by parasite phylogeny

Desdevises *et al*. [1] showed that host specificity in *Lamellodiscus*, monogeneans parasitizing the gills of marine fish of Sparidae, is highly correlated to parasite phylogeny, which suggests that the host specificity of *Lamellodiscus* is influenced by historical constraints. A statistically significant link (but weaker when compared to the *Lamellodiscus* study) between host specificity and parasite phylogeny was also shown for monogeneans of the *Dactylogyrus* genus parasitizing the gills of freshwater Cyprinidae [12]. However, our study revealed that host specificity in *Cichlidogyrus* and *Scutogyrus* species is not linked to parasite phylogeny, suggesting that host specificity is not a phylogenetically conservative feature in cichlid monogeneans. The mapping of the host specificity of parasites onto the phylogenetic tree in both *Lamellodiscus* and *Dactylogyrus* studies [1,12] indicates that strict host specificity (i.e. parasitizing a single host) appears to be the ancestral state for monogeneans and that decreasing host specificity through intermediate specialist, intermediate generalist, and generalist states represents the derived conditions. Nevertheless, our study showed that intermediate specialism (i.e. parasitizing closely related congeneric hosts) represents the ancestral state for gill monogeneans of the *Cichlidogyrus* and *Scutogyrus* group at the global scale. This fact may be explained by the rapid speciation and diversification of cichlid fish on the African continent, generating a wide range of morphologically and ecologically similar, and phylogenetically closely related congeneric cichlid species. Such cichlid congeners tend to be parasitized by the same *Cichlidogyrus* species. Using the mapping of host specificity at a local level, strict specialism was revealed as the ancestral state of host specificity in *Cichlidogyrus* and *Scutogyrus* parasites. The differences between two mappings at two different spatial levels of investigation support the idea of the geographical isolation of congeneric host species parasitized by the same *Cichlidogyrus* species and highlight the problem of the delimitation of host specificity in evolutionary studies. In accordance with previous published studies (e.g. Desdevises *et al*. [1], Šimková *et al*. [12] and Poulin *et al*. [66]) our findings do not support the conventional view, which considers specialization (the process linked to host specificity) as an evolutionary “dead-end” where generalists might evolve into specialists, but not vice versa [67].

### Morphological correlates of host specificity

The morphology of the attachment apparatus in parasites is usually considered to be the result of adaptive processes. It has been assumed that the morphology of the monogenean haptor is connected with host specificity [68]. Šimková *et al*. [21] found that in the case of specialist *Dactylogyrus* parasites, the total length and base length of their anchors were positively correlated with host body size, but no significant relationship was found for generalists. Thus, they suggested that parasite specialization was linked to the adaptation of the haptor to the host. However, Vignon *et al*. [69] found no relationship between host specificity and the morphology of the attachment apparatus in African *Cichlidogyrus* species and pointed out that the attachment apparatus of these parasites exhibits no host-related adaptation. Moreover, they suggested that the morphology of haptoral sclerites reflected parasite phylogeny rather than adaptation to their hosts or environment. Our study may support the findings of Vignon *et al*. [69], as we found only a weak relationship between haptor morphometry and the index of host specificity i.e. only morphometric variables of dorsal anchors and length of 1st pair of marginal hooks were related to host specificity in our study. The majority of the *Cichlidogyrus* species analyzed in our study were collected from the tilapiines (i.e. *Tilapia*, *Oreochromis* and *Sarotherodon*) possessing similar morphology [70], which imposes no selection on the evolution of morphological adaptation in specific cichlid monogenean parasites. The similar morphology of the attachment apparatus may also indicate that the morphological features of the haptors of closely related parasite species are inherited from their common ancestor (we found that many morphometric variables of the haptor are significantly related to parasite phylogeny in specialists and generalists). In this context, intrahost speciation (i.e. parasite duplication) followed by host switching in tilapiines hosts, which was proposed to explain the diversification of *Cichlidogyrus/Scutogyrus* parasites on African cichlids [17], may support this assumption.

Following the hypothesis of ecological specialization [23], first applied to parasites by Morand and Guégan [22], species using more resources (i.e. more host species in the case of parasites) are more abundant and more widespread than species that use a narrow spectrum of sources (i.e. a single host species). In accordance with this hypothesis, Šimková *et al*. [11] analyzing the *Dactylogyrus* communities of roach (*Rutilus rutilus*) showed that generalists of the *Dactylogyrus* species have higher prevalence and reach higher abundance than specialists of the *Dactylogyrus* species. A positive relationship between parasite abundance and the number of host species exploited by a particular parasite species was also found in 22 metazoan parasite species infecting fish of Salmonidae, Cyprinidae, Catostomidae, Centrarchidae and Percidae from the streams of North Carolina [71]. Krasnov *et al*. [72] pointed out that the same properties that enable a parasite species to exploit various environmental conditions and resources also allow it to attain a broad distribution with high local abundance. In contrast with these studies, Poulin [73] found a negative relationship between the number of freshwater fish species and the abundance of their metazoan parasites. This finding may be explained by the high cost of parasite adaptations to multiple hosts, i.e. parasites exploiting a broader host spectrum are forced to invest more in defense mechanisms against a wider range of host species and therefore are not able to attain greater abundance in these hosts [74]. However, our study on gill monogenean parasites of cichlid fishes does not support the hypothesis of ecological specialization, because there was no statistical link between host range and the abundance of *Cichlidogyrus*/*Scutogyrus* species. Nevertheless, in our study (similarly as in the study of Šimková *et al*. [12]), not all host species included in the host range of generalist parasites were analyzed for parasite abundance. Therefore, we suggest that this hypothesis should be re-evaluated by completing data on the abundance of generalist parasites in all host species.

Using monogeneans parasitizing fish, a significant link between host specificity and host body size was found, suggesting that specialists tend to use larger hosts than generalists [1,3,21], which is in line with the hypothesis of specialization on a predictable resource [19]. Larger fish live longer and/or are positioned on top of the food chain [75], and, therefore, represent a more stable habitat, which may favour longer-lived and larger parasites [76]. In addition, larger fish represent more available niches for parasite colonization or specialization and may be more accessible to parasites than smaller fish [1,4,77]. Nevertheless, our study revealed that decreasing host specificity is associated with higher cichlid longevity, indicating that generalist *Cichlidogyrus* tend to colonize long-lived cichlids.

Sasal and Morand [78] showed a significant correlation between host body size and specialist parasite body size in monogeneans parasitizing Mediterranean fishes which is in line with the hypothesis of specialization on predictable resources when considering host body size as a measure of host predictability. In the case of the *Dactylogyrus*-Cyprinidae system, a positive relationship was found between the body size of specialist monogenean parasites and host longevity [12]. Desdevises *et al*. [1] proposed that a significant positive correlation between parasite body size and host size in specialist parasites indicates that host specificity is linked to morphological adaptation. In our study, we failed to find a relationship between host specificity and both host and parasite body sizes. In addition, fish longevity was not related to parasite body size in the context of host specificity, suggesting that body size in specific *Cichlidogyrus* and *Scutogyrus* parasites does not represent potential morphological adaptation. Šimková *et al*. [12] showed that specialists of *Dactylogyrus* associated with longer-lived or larger fish develop large anchors, which was interpreted as a mechanism for optimizing morphological adaptation. However, in the case of generalist *Cichlidogyrus*, the increase in some measures of all sclerotized components of the haptor was positively linked to larger host body size or longevity. Conversely, in the case of specialist *Cichlidogyrus*, a negative relationship was found between two measures of the haptor and cichlid longevity. This may suggest that larger *Cichlidogyrus* specialists tend to occupy mostly short-living host species, whereas *Cichlidogyrus* generalists with large haptor components tend to occupy longer-lived cichlid fish of large body size. Thus, increasing the size of haptor components cannot be viewed as morphological adaptation in specific parasites of *Cichlidogyrus* and *Scutogyrus* genera (see also above). However, the finding concerning fish longevity should be interpreted carefully, because no data on longevity were available for many fish species and in such cases we used data on the longevity of the most closely related congeneric species.

The absence of significant relationships between parasite and host body size may reflect coevolutionary history in the *Cichlidogyrus*/*Scutogyrus*-cichlid system, in which host switching and duplication are considered to be the most important evolutionary events in parasite diversification [17]. In this context, Morand *et al*. [20] hypothesized that if lateral transfers of parasites occur from one host species to another, or parasites duplicate on their hosts, then any change in host body size will have a correlated effect on parasite body size. However, our study is not in the line of this hypothesis. In addition, Poulin [79] suggested that the large size of parasites may have been inherited from a free-living ancestor and is not the result of directional selection toward large size.

Even though our study does not support the hypothesis of parasite specialization on predictable host resources, we showed that parental care behavior is an important life trait in cichlid fish with respect to determining the host specificity of their gill monogenean parasites. Thus, parasite species with higher host specificity tend to select fish species, which exhibit only mouthbrooding behavior or only substrate-brooding behavior, whilst decreasing host specificity is associated with fish hosts exhibiting both forms of parental care. This contradicts Pouyaud *et al*. [28], who claimed that no *Cichlidogyrus* species is able to infect the cichlids with different parental care behavior.

## Conclusions

In conclusion, we showed that the different level of investigation (local vs. global) affects the delimitation of host specificity in *Cichlidogyrus*/*Scutogyrus* parasites. This fact highlights the need to use compiled data on host specificity in evolutionary studies. Our findings do not support the conventional view that specialization is an evolutionary dead-end and support the previous studies investigating the evolution of host specificity in congeneric monogeneans i.e. host specificity in congeneric monogeneans is not a derived condition. However, in contrast to previous studies, the intermediate specialist represents the ancestral character of host specificity in *Cichlidogyrus*/*Scutogyrus* parasites. The haptor morphology (expressed by morphometry) reflected parasite phylogeny rather than adaptation. Our study did not support the specialization on predictable resources or ecological specialization hypotheses. However, we showed that host specificity of *Cichlidogyrus*/*Scutogyrus* species is linked to parental care behavior of cichlid hosts. All these findings may reflect evolutionary history of cichlids that have undergone rapid speciation and diversification on the African continent, and the coevolutionary history in the *Cichlidogyrus*/*Scutogyrus*-cichlid system, in which duplication and host switching are suggested to be the principal coevolutionary events in the diversification of this parasite group.

## Competing interests

The authors declare that they have no competing interests.

## Authors’ contributions

MM performed laboratory analyses and the statistical analyses under the guidance of AS. AS designed the study. MM and AS were involved in drafting the manuscript and approved final version of the manuscript.

## Supplementary Material

Additional file 1 **List of monogenean species used in this study and their African cichlid host species recorded at the global and local levels of investigation.** Host species of parasites at the local level are highlighted by asterisks. Click here for file

Additional file 2 **Data on *Cichlidogyrus *** **and *Scutogyrus *** **species and their hosts used in this study.** Index of host specificity (IS): (1) strict specialist, (2) intermediate specialist, (3) intermediate generalist, (4) generalist. Parental care: (1) mouthbrooder, (2) substrate-brooder, (3) both types of parental care. Click here for file
