# Supplementary material for: Evolution of host specificity in monogeneans parasitizing African cichlid fish
Source: Parasit Vectors. 2014 Feb 14;7:69. doi: 10.1186/1756-3305-7-69 (PMC3932501; doi:10.1186/1756-3305-7-69)
Supplement: Additional file 1 — List of monogenean species used in this study and their African cichlid host species recorded at the global and local levels of investigation. Host species of parasites at the local level are highlighted by asterisks. [file 1756-3305-7-69-S1.doc]

**Additional file 1 List of monogenean species used in this study and their African cichlid host species recorded at the global and local levels of investigation. Host species of parasites at the local level are highlighted by asterisks.**

| Parasite species | Host species |
| --- | --- |
| *Cichlidogyrus acerbus* Dossou, 1982 | *Sarotherodon galilaeus galilaeus*****,*** *S. melanotheron heudelotii, S. m. melanotheron** |
| *Cichlidogyrus aegypticus* Ergens, 1981 | *Tilapia busumana, T. camerunensis, T. dageti, T. guineensis*, T. gutturosa, T. kottae, T. louka, T. walteri, T. zillii* |
| *Cichlidogyrus agnesi* Pariselle & Euzet, 1995 | *Tilapia guineensis** |
| *Cichlidogyrus amphoratus* Pariselle & Euzet, 1996 | *Tilapia guineensis*, T. louka* |
| *Cichlidogyrus arthracanthus* Paperna, 1960 | *Tilapia busumana, T. camerunensis, T. dageti, T. deckerti, T. guineensis*, T. gutturosa, T. kottae, T. walteri, T. zillii* |
| *Cichlidogyrus bilongi* Pariselle & Euzet, 1995 | *Tilapia camerunensis, T. guineensis** |
| *Cichlidogyrus cirratus* Paperna, 1964 | *Oreochromis esculentus, O. niloticus niloticus*, O. variabilis, Sarotherodon galilaeus galilaeus*, Tilapia zillii* |
| *Cichlidogyrus cubitus* Dossou, 1982 | *Tilapia busumana, T. buttikoferi, T. camerunensis, T. dageti, T. guineensis*, T. louka, T. mariae, T. walteri, T. zillii* |
| *Cichlidogyrus digitatus* Dossou, 1982 | *Tilapia brevimanus, T. camerunensis, T. dageti*, T. guineensis*, T. louka, T. mariae, T. walteri, T. zillii** |
| *Cichlidogyrus douellouae* Pariselle, Bilong Bilong & Euzet, 2003 | *Sarotherodon galilaeus galilaeus*, S. g. sanagaensis* |
| *Cichlidogyrus* *dracolemma* Řehulková, Mendlová & Šimková, 2013 | *Hemichromis letourneuxi** |
| *Cichlidogyrus ergensi* Dossou, 1982 | *Tilapia camerunensis, T. dageti, T. guineensis*, T. louka, T. mariae, T. walteri, T. zillii* |
| *Cichlidogyrus falcifer* Dossou  Birgi, 1984 | *Hemichromis fasciatus** |
| *Cichlidogyrus flexicolpos* Pariselle & Euzet, 1995 | *Tilapia dageti*, T. guineensis*, T. mariae* |
| *Cichlidogyrus gallus* Pariselle & Euzet, 1995 | *Tilapia guineensis*, T. walteri, T. zillii* |
| *Cichlidogyrus halli* (Price & Kirk, 1967) | *Oreochromis aureus, O. esculentus, O. leucostictus, O. mortimeri, O. mossambicus, O. niloticus niloticus, O. niloticus vulcani, O. shiranus chilwae, O. shiranus shiranus, O. spirulus spirulus, O. variabilis, Sarotherodon galilaeus galilaeus*, S. melanotheron melanotheron, S. occidentalis, Serranochromis macrocephalus, Tilapia guineensis*, T. zillii* |
| *Cichlidogyrus longicirrus* Paperna, 1965 | *Hemichromis fasciatus*, Chromidotilapia guentheri* |
| *Cichlidogyrus* *nageus* Řehulková, Mendlová & Šimková, 2013 | *Sarotherodon galilaeus galilaeus*, Tilapia guineensis** |
| *Cichlidogyrus njinei* Pariselle, Bilong Bilong & Euzet, 2003 | *Sarotherodon galilaeus galilaeus*, S. g. sanagaensis, Tilapia guineensis** |
| *Cichlidogyrus pouyaudi* Pariselle & Euzet, 1994 | *Tylochromis intermedius*, T. jentinki* |
| *Cichlidogyrus sclerosus* Paperna  & Thurston, 1969 | *Haplochromis* sp., *Oreochromis aureus, O. leucostictus, O. mortimeri, O. mossambicus, O. niloticus niloticus, O. spirulus niger, Serranochromis macrocephalus, Tilapia zillii* |
| *Cichlidogyrus thurstonae* Ergens, 1981 | *Haplochromis longirostris, Oreochromis aureus, O. esculentus, O. mossambicus, O. niloticus niloticus*, O. variabilis, Sarotherodon galilaeus galilaeus* |
| *Cichlidogyrus tiberianus* Paperna, 1960 | *Haplochromis flavijosephi, Tilapia bakossiorum, T. busumana, T. coffea, T. dageti*, T. guineensis*, T. gutturosa, T. kottae, T. mariae, T. rendalli, T. walteri, T. zillii** |
| *Cichlidogyrus tilapiae* Paperna, 1960 | *Haplochromis macrognathus, Hemichromis fasciatus*, Chromidotilapia guentheri, Oreochromis aureus, O. leucostictus, O. mortimeri, O. mossambicus, O. niloticus niloticus*, O. niloticus vulcani, O. spirulus niger, O. variabilis, O. urolepis urolepis, Sarotherodon galilaeus galilaeus*, Tilapia busumana, T. buttikoferi, T. camerunensis, T. guineensis*, T. gutturosa, T. kottae, T. mariae, T. zillii* |
| *Cichlidogyrus yanni* Pariselle & Euzet, 1996 | *Tilapia camerunensis, T. dageti*, T. guineensis*, T. louka, T. mariae, T. walteri, T. zillii* |
| *Scutogyrus bailloni* Pariselle & Euzet, 1995 | *Sarotherodon galilaeus galilaeus** |
| *Scutogyrus longicornis* Paperna  & Thurston, 1969 | *Oreochromis aureus*, O. niloticus niloticus*, O. mortimeri, O. mosambicus, Sarotherodon galilaeus galilaeus, S. melanotheron melanotheron* |
| *Scutogyrus minus* (Dossou, 1982) | *Oreochromis niloticus niloticus*, *Sarotherodon melanotheron melanotheron* |
