# Supplementary material for: Evolution of host specificity in monogeneans parasitizing African cichlid fish
Source: Parasit Vectors. 2014 Feb 14;7:69. doi: 10.1186/1756-3305-7-69 (PMC3932501; doi:10.1186/1756-3305-7-69)
Supplement: Additional file 2 — Data on Cichlidogyrus and Scutogyrus species and their hosts used in this study. Index of host specificity (IS): (1) strict specialist, (2) intermediate specialist, (3) intermediate generalist, (4) generalist. Parental care: (1) mouthbrooder, (2) substrate-brooder, (3) both types of parental care. [file 1756-3305-7-69-S2.doc]

**Additional file 2 Data on *Cichlidogyrus* and *Scutogyrus* species and their hosts used in this study. Index of host specificity (IS): (1) strict specialist, (2) intermediate specialist, (3) intermediate generalist, (4) generalist. Parental care: (1) mouthbrooder, (2) substrate-brooder, (3) both types of parental care.**

| Parasite species | Abundance | Host range | IS global | IS local | Maximum parasite body size (in µm)  (in μm) | Host body  size (in cm) | Longevity | Parental care |
| --- | --- | --- | --- | --- | --- | --- | --- | --- |
| *C. acerbus* | 0.17 | 3 | 2 | 2 | 650 | 28.3 | 7 | 1 |
| *C. aegypticus* | 0.96 | 9 | 2 | 1 | 782 | 24.3 | 7 | 2 |
| *C. agnesi* |  | 1 | 1 | 1 | 954 | 30 | 7 | 2 |
| *C. amphoratus* | 1.73 | 2 | 2 | 1 | 686 | 26 | 7 | 2 |
| *C. arthracanthus* | 1.85 | 9 | 2 | 1 | 1170 | 24.7 | 7 | 2 |
| *C. bilongi* |  | 2 | 2 | 1 | 1052 | 30 | 7 | 2 |
| *C. cirratus* | 21.25 | 5 | 3 | 3 | 1090 | 42.8 | 8.7 | 3 |
| *C. cubitus* | 0.28 | 9 | 2 | 1 | 580 | 26.9 | 7 | 2 |
| *C. digitatus* | 8.32 | 8 | 2 | 2 | 460 | 28.5 | 7 | 2 |
| *C. douellouae* | 0.58 | 2 | 2 | 1 | 735 | 23.6 | 7 | 1 |
| *C. dracolemma* | 2.56 | 1 | 1 | 1 | 550 | 11.9 | 10 | 2 |
| *C. ergensi* | 0.8 | 7 | 2 | 1 | 500 | 29.2 | 7 | 2 |
| *C. falcifer* | 1.63 | 1 | 1 | 1 | 550 | 20.4 | 10 | 2 |
| *C. flexicolpos* |  | 3 | 2 | 2 | 760 | 30.5 | 7 | 2 |
| *C. gallus* |  | 3 | 2 | 1 | 696 | 31 | 7 | 2 |
| *C. halli* | 2.43 | 17 | 4 | 3 | 721 | 37.4 | 8.5 | 3 |
| *C. longicirrus* | 5 | 2 | 4 | 1 | 500 | 17.5 | 10 | 3 |
| *C. nageus* | 0.32 | 2 | 3 | 3 | 576 | 32 | 7 | 3 |
| *C. njinei* | 1.08 | 3 | 3 | 3 | 760 | 25.7 | 7 | 3 |
| *C. pouyaudi* | 3.19 | 2 | 2 | 1 | 410 | 25 | 7 | 1 |
| *C. sclerosus* |  | 9 | 4 |  | 1400 | 39.9 | 8.2 | 3 |
| *C. thurstonae* |  | 7 | 4 | 1 | 835 | 37.8 | 10 | 1 |
| *C. tiberianus* | 0.81 | 12 | 4 | 2 | 700 | 20.7 | 7 | 3 |
| *C. tilapiae* | 0.77 | 21 | 4 | 4 | 500 | 29.9 | 8 | 3 |
| *C. yanni* | 0.48 | 7 | 2 | 2 | 764 | 27.7 | 7 | 2 |
| *S. bailloni* | 0.08 | 1 | 1 | 1 | 1114 | 34 | 7 | 1 |
| *S. longicornis* | 1.5 | 6 | 3 | 2 | 971 | 39.6 | 9.3 | 1 |
| *S. minus* |  | 2 | 3 |  | 884 | 44 | 9 | 1 |
